# “Characteristics of patients admitted to emergency department for asthma attack: a real-LIFE study”

**DOI:** 10.1186/s12890-019-0869-8

**Published:** 2019-06-17

**Authors:** Laura Losappio, Enrico Heffler, Rossella Carpentiere, Monica Fornero, Cosimo Damiano Cannito, Francesco Guerrera, Francesca Puggioni, Riccardo Monti, Stefania Nicola, Giovanni Rolla, Luisa Brussino

**Affiliations:** 1 Allergy and Immunology Department, Niguarda Ca Granda Hospital, Milan, Italy; 2 Personalized Medicine, Asthma and Allergy, Humanitas Research Hospital, Rozzano, Italy; 3 Department of Biomedical Sciences, Humanitas University, Rozzano, Italy; 4 Emergency Department, “Dimiccoli” Hospital, Barletta, Italy; 5 Department of Surgical Science, University of Torino, Torino, Italy; 6 Allergy and Clinical Immunology Unit, Department of Medical Science, University of Torino and AO Ordine Mauriziano Umberto I, Torino, Italy

**Keywords:** Asthma attack, Asthma therapy, Adult asthma, Allergic asthma, Anti-asthmatic drugs

## Abstract

**Background:**

Asthma is a chronic disease affecting 30 million people in Europe under 45y. Poor control of Asthma is the main cause of emergency-department (ED) access, becoming the strongest determinant of the economic burden of asthma management.

**Objective:**

To examine the characteristics of adult patients admitted to ED for acute asthma attack, focusing on previous diagnosis of asthma (DA) and current therapy.

**Methods:**

During a one-year period, a structured questionnaire, assessing asthma diagnosis and management, was administered to all patients admitted for asthma attack, to the ED of a South-Italy town. Only patients with subsequently confirmed asthma were enrolled.

The data on oxygen saturation (Sat.O2), heart and respiratory-rate, severity code ED-admission, hospitalization or discharge, had been obtained.

**Results:**

Two hundred one patients (mean 50.3ys), were enrolled. One hundred eighteen had a DA, made 17.5 ± 5.88 years before, and 35.6% had a specialist-examination in the last year. 53.3% of DA-patients used a self-medication before ED access with short-acting-beta-2-agonist and oral-corticosteroids, although none had a written-asthma-action-plan (WAAP). Almost all DA-patients were on regular therapy: inhaled-corticosteroids (ICS) in 61%, associated with LABA in 85%. 16.7% of DA-patients had previous DA-access. The overall hospitalization-rate was 39%, higher in DA compared to unknown asthmatic patients (UA)(*p* = 0.017).

Significant risk factors for hospitalization were Sat-O2 ≤ 94% breathing ambient air (OR9.91, *p* < 0.001), inability-to-complete a sentence (OR9.42,p < 0.001) and the age (OR1.02,*p* = 0.049).

**Conclusion:**

Despite the asthma guidelines-recommendation, up to 40% of patients received the asthma diagnosis in ED, only 61% of DA-patients were taking ICS. It is disappointing that DA-patients did not have a WAAP, which could explain the poor patient-self-medication at ED admission.

**Electronic supplementary material:**

The online version of this article (10.1186/s12890-019-0869-8) contains supplementary material, which is available to authorized users.

## Background

Asthma is an important public health problem in Europe, affecting around 30 million children and adults under 45 years of age [1]. Estimates indicate that the age standardized hospital admission rate for asthma ranges from 30 to 70 per 100.000 Europeans older than 15 years, with an estimated economic burden of € 19.5 billion annually [1, 2]. The lack of disease control has been reported to be the strongest determinant of the individual total cost, which was 3-fold higher among uncontrolled subjects compared to controlled/partly controlled individuals, due to the increase in the indirect and hospital costs [3, 4]. Emergency department visits impose a heavy economic burden on health care, as each emergency visit costs 5-fold more than outpatient visits for asthma [5]. Unfortunately, epidemiological surveys suggest that the control of asthma is still poor in the general population, mainly due to under-treatment of the disease [6–8].

Most emergency department (ED) visits for acute asthma are thought to be preventable [9], considering that the management of ED frequent attenders was found suboptimal according to guidelines [10].

Despite the heavy economic burden of asthma-related ED visits, few studies have been designed to characterize the asthmatic patients who attend ED for acute asthma. According to a large epidemiological study, primarily focused on the quality of emergency asthma care, the proportion of asthmatic patients taking inhaled corticosteroids as long-term control medication was only 35% [11] and although the guidelines recommend the prescription of written asthma action plans (WAAP), their use remains limited [12, 13]. The aim of the study was to examine the patient characteristics of adult patients admitted to the Emergency Department of Dimiccoli Hospital (the general hospital of Barletta, a 90,000 inhabitants town of South Italy) for an acute asthma attack, focusing on previous diagnosis of asthma and current asthma therapy.

## Methods

All patients (age ≥ 12 and < 65 years) admitted to the ED for suspected acute asthma attack (ICD-9-CM 2012 Diagnosis Code: 493 as primary diagnosis) since 1st January 2013 until 31st December 2013 signed an informed consent form to participate in this study, which had been approved by the Local Ethic Committee.

A questionnaire was administered to all enrolled patients to obtain information about previous physician diagnosed asthma (DA) and current asthma medications, allergic rhinitis, outpatient visits for asthma and spirometry performed in the last 12 months, previous ED visits for asthma attack in the last 12 months, details of the medication used for asthma attack and whether the patients had received a written asthma action plan (WAAP) and comorbidities including allergic rhinitis were also assessed (Additional file 1). The duration of symptoms before the ED access was also considered. In this regard, the time lapse between the onset of the asthma attack and the ED attendance has been partitioned up to 6 h, 6 to 24 h and more than 24 h. Patients who had not previously received a diagnosis of asthma or who were not taking any medication for asthma, including inhalers, aerosol or tablets, were classified as possible unknown asthma. Every possible unknown asthmatic enrolled was evaluated by an Allergist/Pneumologist in the following months and was included in the final study population only if the diagnosis of asthma had been confirmed, according to GINA guidelines. These patients have been classified as “Unknown Asthma” (UA) at the ED admission.

Data on oxygen saturation, heart and respiratory rate, severity code admission at ED, ED course, and ED disposition about hospitalization or discharge, were obtained by chart review.

Statistical analysis was performed using a commercially available statistical package Stata 13.1 (Stata Corp LP, College Station, TX, USA), considering statistically significant only *p* values < 0.05. Data are given as means ± SD.

Three normality tests were performed (Kolmogorov-Smirnov, Shapiro-Wilk and D’Agostino‘s K-squared), to establish the distribution of the data. Basing on the normal or not normal distribution, the comparisons between clinical data from UA and DA patients, were performed using Student’s t-test or Mann-Whitney U test.

The factors associated with a patient’s admission were assessed with the use of logistic regression analysis. All factors associated with *p* < 0.10 on univariable analysis were included in the multivariate model. All tests were two-sided, and variables were considered significant for *p*-values < 0.05.

## Results

Two hundred one patients had been admitted to the ED in 12 months, 126 (62.7%) male and 75 (37.3%) female, with a mean age 50.3 years (range 12–65 yrs).

Of all the patients who were included, there were 118 (58.7%) with DA and 83 (41.3%) with UA. Clinical and demographic characteristics of the two groups are reported in Table 1.

**Table 1:** **-** Clinical and demographic characteristics of the enrolled patients with previously diagnosed (DA) and not previously known (UA) asthma

Characteristics	DA *n* = 118	UA *n* = 83	p
Male %	61	65	ns
Mean age (range)	50.3 (12–64)	46.4 (12–65)	ns
Specialist examination in the last 12 months n (%)	42 (35.6)	–	
Rhinitis n (%)	64 (54,2)	5 (6)	p < 0.001

Male %: percentage of male enrolled to the study. Mean age (range): mean age of people enrolled to the study. Specialist examination in the last 12 months: number of people who consulted a specialist in the last 12 months. Rhinitis n (%): percentage of people affected by rhinitis

In the group of DA patients, the diagnosis had been made 17,5 (95%CI 14.8–19.9) years before, and 42 out of 118 (35.6%) had a specialist examination in the last 12 months. Allergic rhinitis was reported by 54.2% of patients with DA and by 6% of patients with UA (*p* < 0.001). Concerning the time lapse between the onset of asthma attack and the ED access, a greater prevalence of ED access between the 6th and 24th hour was observed in DA patients (62.7%) compared to UA patients (44.6%, *p* = 0.011), while no differences were observed in early access (< 6 h), respectively 21,2 and 30,2%, and in late access (> 24 h), respectively 12.7 and 20.5%.

Sixty-three DA patients (53.3% of all DA patients) used self-medication before the ED access, consisting of short-acting beta-2 agonist (SABA) in 57 of them (90%) and oral corticosteroids in 6 (10%), according to their physicians’ advice. No patient had a WAAP and none used peak flow assessment.

Almost all the DA patients (112/118, 94.9%) were on regular therapy, consisting of inhaled corticosteroids (ICS) in 68/112 (60.71%), associated with LABA in 85% of them, SABA alone in 14 patients (12.5%), antihistamines for associated allergic rhinitis in 25 (22%), oral corticosteroids (OCS) in 4 (3,5%) and theophylline in one patient (0,9%).

Twenty DA patients (16.9%) had an history of previous ED access during the last 12 months. Among these patients, 14 (70%) had at least one specialist examination in the last 12 months, 18 (90%) received regular treatment with ICS, combined with LABA in 78%, while two patients (10%) were taking only antihistamine drugs for concomitant rhinitis.

The hospitalization rate after acute admission in ED was 38.8% (78/201) for the whole study population, significantly higher in DA compared to UA patients (64 and 36% respectively, *p* = 0.017).

Clinical and demographic characteristics of hospitalized patients (HP) compared to patients who were discharged are reported in Table 2. Patients who were hospitalized were older (60.8 vs 43.5 yrs., *p* < 0.001), had higher prevalence of previous diagnosis of asthma (64.1 vs 55%, *p* = 0.009) and previous ED visits (16.7% vs 11.4%, p = 0.01), lower SaO2 (89.8 vs 96.1%, p < 0.001), higher respiratory rate (22 ± 4 vs 18.5 ± 2.8, p < 0.001) and pulse rate (85.4 ± 15 vs 80.3 ± 13, *p* = 0.004), and they were more frequently unable to complete a sentence (67.95 vs 13.8%, p < 0.001).

**Table 2:** – Risk factors determining the hospitalization (201 patients)

	Hospitalized patients (n 78)	Discharged patients (n 123)	p =
Age - years (range)	60.8 (12–65)	43.5 (12–63)	< 0.001
DA (%)	64.1	55	0.009
≥1 ED admission (%)	16.7	11.4	0.010
SaO2% (SD)	89.8 (8.09)	96.1 (2.74)	< 0.001
Respiratory rate (SD)	22 (3.97)	18.53 (2.76)	< 0.001
Pulse rate (SD)	85.4 (15.01)	80.33 (13.04)	0.004
Years from diagnosis (SD)	14.53 (5.88)	7.41 (11.26)	Ns
ED admission code (%)	Green 15.4% Yellow 64.1% Red 20.5%	Green 57.7% Yellow 42.3%	< 0.001
Inability to complete a sentence (%)	67.95	13.82	< 0.001

Age - yrs. (range): mean age of the two considered groups. DA (%): percentage of patients with previous asthma diagnosis. ≥1 ED admission (%): percentage of patients with more than one ED admission in the last 12 months. SaO2% (SD): mean pulse oximetry at ED visit. Respiratory rate (SD): mean values of respiratory rate at ED visit. Pulse rate (SD): mean values of pulse rate at ED visit. Years from diagnosis (SD): numbers of years since the diagnosis of asthma. ED admission code (%): index of disease severity (green: low severity; yellow: medium severity; red: emergency). Inability to complete a sentence (%): percentage of people who could not complete a sentence due to dyspnea

Multivariate analysis (Table 3) showed that independent significant risk factors for hospitalization were oxygen saturation lower than 94% at breathing air (OR 9.91, *p* < 0.0001), the inability to complete a sentence (OR 9.42, p < 0.0001) and the age of the patients (OR 1.02, *p* = 0.049).

**Table 3:** - Multivariate analysis of risk factor affecting the hospitalization (193 Patients)

Variable	Odd Ratio [95%CI]	p =
Age	1.02 [1.01–1.05]	0.049
Years from diagnosis (SD)	1.00 [0.97–1.05]	ns
Time lapse< 6 h	0.80 [0.28–2.33]	ns
6–24 h	0.81 [0.29–2.31]	ns
> 24 h	1.2 [0.31–4.74]	ns
SaO2 ≤ 94	9.91 [3.83–25.6]	< 0.0001
Respiratory rate ≥ 18/min	2.99 [0.78–11.5]	ns
Inability to complete a sentence	9.42 [3.60–24.7]	< 0.0001

Age: mean age of patients hospitalized or not. Lenght from Diagnosis: years since diagnosis of asthma. Time lapse < 6 h: less than 6 h between symptoms onset and ED admission. 6–24 h: 6 to 24 h between symptoms onset and ED admission. > 24 h: more than 24 h between symptoms onset and ED admission. SaO2 < =94: pulse oximetry at ED visit less than 94% on air (FiO2 21%). Respiratory rate ≥ 18/min: respiratory rate more than 18 acts per minute

## Discussion

A surprising result of our study was that 40% of patients presenting to the ED received the diagnosis of asthma for the first time. It is known that asthma may be diagnosed for the first time in a patient presenting to the ED, but, in the majority of cases, the patient will be aware of the underlying diagnosis of asthma [14].

A confident diagnosis of asthma cannot be made on a single visit to the ED, (particularly in children and in older patients, who were not enrolled in our study) and the mis-diagnosis of asthma is also frequent in patients managed in primary care settings [15, 16]. Many conditions that may mimic asthma acutely had been considered and excluded in our patients, such as pulmonary embolism, heart failure or hyperventilation syndrome. In all these patients the diagnosis of asthma was confirmed by a specialist during the follow-up visits after Hospital discharge. It appears that, for many patients, asthmatic symptoms are not considered serious enough to be reported to their general practitioner, until an acute asthma attack occurs, which forces them to go to the emergency department. The only difference between patients with UA compared to patients with DA was the prevalence of rhinitis, which was reported only in 6% of the UA patients compared to 54% of DA. Rhinitis comorbidity may lead the patients with asthmatic symptoms to be evaluated by their physicians for asthmatic symptoms earlier than the patients without rhinitis.

Inhaled corticosteroids were regularly used by 60% of our patients with DA, a percentage higher than the percentage previously reported in patients admitted to ED for asthma, which had been reported as low as 35% in a US large epidemiological study primarily focused on the quality of emergency asthma care during exacerbation [11]. Indeed, despite an increase in the consumption of inhaled corticosteroids (ICS), their use is still inadequate, since five out of 10 asthmatic patients were using a medication regimen below their disease severity level, according to an Italian epidemiologic study and the inadequate dosing of ICS was found to be the main predictor of the poor control of the disease in the same study [6].

High use of health services, including ED visits, has been observed in patients with suboptimal asthma drug regimens in a Canadian population-based assessment study [17]. In particular, adolescents with suboptimal regimens were the most likely to have hospital admissions (odds ratio (OR) 3.8; 95% confidence interval (CI) 1.8–7.8), visit the ED (OR 2.2; 95% CI 1.6–3.1) and be high users of family physician services (OR 5.7; 95% CI 4.0–8.1) compared with patients in other age groups. Recently the diagnosis of asthma has been reported in 12.7% of patients who presented to an Emergency Department in the Asia Pacific region with a principal symptom of dyspnea [18]. Over 90% of these patients had received a previous diagnosis of asthma, but only 40% of them used inhaled corticosteroids as regular treatment. In our study, up to 90% of the patients who had been previously admitted to ED for asthma exacerbation used ICS and most of them had had a specialist visit for their asthma in the last 12 months, compared to only 35% of the other patients. In our patients the reasons for frequently attending ED seemed to be found in the severity of asthma and not in socioeconomic factors, as reported in a US study [19]. It is disappointing that no patient with DA had a written asthma action plan for managing asthma exacerbation. This explains why only 60% of the patients used self-administered medication before their admission at ED.

In conclusion, despite guideline recommendations about asthma diagnosis and treatment, up to 40% of patients presenting to the ED received the diagnosis of asthma for the first time, and ICS were regularly used only by 60% of patients with known asthma. Moreover, it is disappointing that none of the patients had a WAAP. This could explain why only 53% of the patients used self- administered medication before their attendance at the ED.

## Conclusions

Despite asthma guidelines-recommendation, up to 40% of patients received the asthma diagnosis in ED, only 61% of DA-patients were taking ICS. It is disappointing that DA-patients did not have a WAAP, which could explain the poor patient-self-medication at ED admission.

## Additional file

Additional file 1: “Asthma questionnaire – Emergency Department admission due to asthma attack” Description of data: the questionnaire contains data assessing asthma diagnosis and management, primary vital signs and drugs administered in Emergency Department (ED) in patients admitted to the ED for an acute asthma attack. (DOCX 15 kb)

## Data Availability

The datasets generated and analyzed during the current study are not publicly available due to the uncomplete use of them and to the possibility of further studies, but are available from the corresponding author on reasonable request.

## Abbreviations

CI: Confidence Interval
DA: Previous Diagnosis Of Asthma
ED: Emergency-Department
GINA: Global Initiative For Asthma
HP: Hospitalized Patients
ICS: Inhaled-Corticosteroids
LABA: Long Acting Beta 2 Agonists
OR: Odd Ratio
SABA: Short-Acting Beta-2 Agonist
Sat.O2: Oxygen Saturation
UA: Undiagnosed Asthma
WAAP: Written-Asthma-Action-Plan
