# Supplementary material for: “Characteristics of patients admitted to emergency department for asthma attack: a real-LIFE study”
Source: BMC Pulm Med. 2019 Jun 17;19:107. doi: 10.1186/s12890-019-0869-8 (PMC6580601; doi:10.1186/s12890-019-0869-8)
Supplement: Supplementary file 1 — “Asthma questionnaire – Emergency Department admission due to asthma attack” Description of data: the questionnaire contains data assessing asthma diagnosis and management, primary vital signs and drugs administered in Emergency Department (ED) in patients admitted to the ED for an acute asthma attack. (DOCX 15 kb) [file 12890_2019_869_MOESM1_ESM.docx]

**Asthma questionnaire – Emergency Department admission due to asthma attack**

*Inclusion criteria:* Patient with Emergency Department admission due to asthma attack

Emergency Department admission date: ­____/____/______ Time ____:_____

Patient ID: ___________________­__________

Date of birth: ____/____/______ Sex: M □ F □

1. Have you ever been diagnosed with asthma? YES □ NO □

If you answered yes, since when do you suffer from asthma? ________________________

1. Do you know if you are affected by allergic asthma? YES □ NO □
   If you answered yes, what are you allergic to? ____________________________________
2. Do you also suffer from rhinitis? YES □ NO □
3. About the current asthma attack: how many hours before the admission to Emergency Department did it occur?

□ < 6 hours ago □ between 6 and 24 hours ago □ >24 hours ago

1. Do you usually take drugs for asthma? YES □ NO □

If you answered yes, which drugs do you usually take? ________________________

1. Are you regularly followed for asthma by a specialist? YES □ NO □
2. How long ago did you undergo your last specialist visit for asthma? ______________________
3. Did you ever undergo a lung function test? YES □ NO □
   If you answered yes, when did you perform the last spirometry? ___________________________
4. Over the last 12 hours, before your Emergency Department admission, which drugs have you taken?

___________________­_____________________________­____________________________­____

1. Do you have a Written Asthma Action Plan, indicating the drugs you have to take in case of asthma attack? YES □ NO □
2. Have you ever gone to Emergency Department for asthma attacks in the past? YES □ NO □

If you answered yes, how many times over the last year? __________________________________

**To be completed by the clinician:**

1. Asthma severity evaluation:
   1. O2 saturation in breathing air:_____
   2. Respiratory rate: _____________
   3. Heart rate: _________
   4. Patient’s ability to complete a sentence: YES □ NO □
2. Drugs administered in Emergency Department: _______________________________________________________
3. Emergency Department discharge YES □ NO □

If yes, at which time: _________________

1. Hospitalization: YES □ NO □
